# A framework for nurturing doctors: systematic review of wellbeing interventions in medical students

**DOI:** 10.1192/bjo.2021.641

**Published:** 2021-06-18

**Authors:** Rhian Bradley, Tahmina Yousofi, Rafey Faruqui, Kate Hamilton-West

**Affiliations:** 1 Kent and Medway NHS and Social Care Partnership Trust; 2 University of Kent

## Abstract

**Aims:**

UK medical students report high levels of stress, in particular within the coronavirus pandemic: 46% have a probable psychiatric disorder; almost 15% consider suicide; 80% describe support as poor or moderately adequate. Our aim was to propose a novel conceptual framework for the implementation of effective interventions to reduce their stress and support wellbeing.

**Method:**

A systematic review of MEDLINE, PsycINFO and CINAHL databases was undertaken with appropriate search terms, supplemented by reference searching. Published quantitative and qualitative primary research was included. Findings were reported in line with Preferred Reporting Items for Systematic Reviews and MetaAnalyses.

**Result:**

Records identified through database searching 2,347; additional records 139; records following removal of duplicates 1,324. Full text studies included 41: ‘Curriculum and Grading’ (n = 4); ‘Mindfulness and Yoga’ (n = 11); ‘Stress Management/Relaxation’ (n = 13); ‘Behavioural Interventions’ (n = 3); ‘Cognitive & Self-awareness Interventions’ (n = 2); Mentorship (n = 3); ‘Education, Screening and Access to care’ (n = 3); ‘Multifaceted Interventions’ (n = 2).

Effective interventions include those that reduce academic stress through grading changes and supporting transition to clinical training; resilience enhancing interventions such as mindfulness, yoga, CBT, group based exercise and relaxation; peer mentorship; faculty mentorship when actively engaged by the mentor; reducing stigma; improving detection; and improving access to treatment.

Outcomes for clinical year students were less promising, suggesting interventions may be insufficient to combat clinical stressors.

**Conclusion:**

We propose a framework for implementing these effective interventions through ‘Ecological and Preventative’ paradigms. The former highlights an individual's interaction with their sociocultural environment, recognising multiple levels of influence on health: individual, interpersonal, institutional, community, and national. At each level the framework of primary, secondary and tertiary prevention can be applied.

Primary Prevention (intervening before health is impacted): reducing academic stress; resilience interventions; mentorship; peer support; brief interventions to avoid progress to established disorders.

Secondary Prevention (reducing prevalence of disorder): early detection through staff training and screening; treatment referral pathways; reciprocal arrangements if peers are placed within local settings.

Tertiary Prevention (reducing impairment): reasonable adjustments, communicated between placements

This recognises that medical students require a range of interventions at multiple levels to reduce stress, promote wellbeing and manage the spectrum of mental health difficulties they may encounter. The ecological framework also acknowledges the reciprocity of individuals being influenced by and influencing their environment, which aligns with the concept of co-production.

